# Acute exercise and aerobic fitness influence selective attention during visual search

**DOI:** 10.3389/fpsyg.2014.01290

**Published:** 2014-11-11

**Authors:** Tom Bullock, Barry Giesbrecht

**Affiliations:** ^1^UCSB Attention Lab, Department of Psychological and Brain Sciences, University of CaliforniaSanta Barbara, CA, USA; ^2^Institute for Collaborative Biotechnologies, University of CaliforniaSanta Barbara, CA, USA

**Keywords:** attention, visual search, distraction, physical activity, exercise, fatigue, aerobic fitness, VO_2_max

## Abstract

Successful goal directed behavior relies on a human attention system that is flexible and able to adapt to different conditions of physiological stress. However, the effects of physical activity on multiple aspects of selective attention and whether these effects are mediated by aerobic capacity, remains unclear. The aim of the present study was to investigate the effects of a prolonged bout of physical activity on visual search performance and perceptual distraction. Two groups of participants completed a hybrid visual search flanker/response competition task in an initial baseline session and then at 17-min intervals over a 2 h 16 min test period. Participants assigned to the exercise group engaged in steady-state aerobic exercise between completing blocks of the visual task, whereas participants assigned to the control group rested in between blocks. The key result was a correlation between individual differences in aerobic capacity and visual search performance, such that those individuals that were more fit performed the search task more quickly. Critically, this relationship only emerged in the exercise group after the physical activity had begun. The relationship was not present in either group at baseline and never emerged in the control group during the test period, suggesting that under these task demands, aerobic capacity may be an important determinant of visual search performance under physical stress. The results enhance current understanding about the relationship between exercise and cognition, and also inform current models of selective attention.

## INTRODUCTION

One essential feature of the human attention system is the ability to selectively process goal-relevant visual information while ignoring goal-irrelevant information. Coherent behavior in our complex environment requires a flexible selective attention system that can not only adapt to changes in perceptual and cognitive task demands, but also to additional challenges caused by physical fatigue and stress. Current models of selective attention, such as perceptual load theory (Lavie, 1995; Lavie et al., 2004) and the theory of visual attention (TVA; Bundesen, 1990; Bundesen et al., 2005) have focused on the flexibility of selective attention in response to changes in task demands and goals in tightly controlled laboratory studies carried out under conditions of low stress. What is largely unclear from this work, however, is the extent to which physical fatigue modulates selective attention. The aim of the present work is to gain a more comprehensive understanding of the effects of long bouts of physical activity on the functioning of the attention system during visual search.

Brief, acute bouts of physical activity can influence performance in a range of cognitive tasks (for meta-analytical reviews see Lambourne and Tomporowski, 2010; Chang et al., 2012). Several physiological mechanisms are thought to contribute to the effects of exercise-induced arousal on cognition, including changes in heart rate (e.g., Hillman et al., 2003; Davranche et al., 2005) and levels of brain-derived neurotrophic factor (BDNF; Ferris et al., 2007). A number of studies have investigated exercise effects on specific aspects of selective attention and cognitive control, but the findings have been inconsistent. For example, relative to visual search performed at rest, the time to detect a target object amongst distractors during visual search is faster during an acute bout of exercise at 100% of maximum aerobic capacity (McMorris and Graydon, 1997) and after a 10 min bout of cycling at up to 85% of maximum aerobic capacity (Aks, 1998). In contrast, Bard and Fleury (1978) reported no difference in visual search target detection speed in participants tested before and after a maximal VO_2_max test. Similarly, in flanker response competition tasks (e.g., Eriksen and Eriksen, 1974) there is also evidence of a response time benefit (i.e., speeding up) during a 20 min bout of exercise at 50% of maximal aerobic workload that is independent of the level of distractor interference (Davranche et al., 2009). However, Hillman et al. (2003) found no effect of a 30 min bout of treadmill running at >80% of maximum heart rate on RTs in a flanker task, although they did find neural evidence from EEG to suggest enhanced stimulus classification speed and a relationship between individual aerobic capacity, neural, and behavioral indices of error monitoring (Themanson and Hillman, 2006). This discrepant pattern of results may have many causes, including differences in exercise type, differences in exercise intensity and duration, the design of the behavioral task and the timing of its administration, and individual aerobic capacity and experience with exercise.

Despite the conflicting evidence in the literature, it is clear that relatively brief bouts of acute exercise can influence multiple aspects of selective attention and cognitive control. However, few studies have tested the impact of extended bouts of activity on cognition and, as a result, the effects of these extended bouts are less well understood. Prolonged exercise can lead to hypoglycemia, which can result in increased central fatigue and increased ratings of perceived exertion because the supply of metabolites to the brain is restricted (Nybo and Secher, 2004). Furthermore, acute hypoglycemia induced via insulin infusion can have a detrimental effect on cognitive performance (Strachan et al., 2001; Schachinger, 2003). While it is reasonable to infer that hypoglycemia associated with a long bout of physical activity may also impact cognitive function, there is limited empirical evidence for this in the literature and the evidence that does exist is contradictory. For example, Hogervorst et al. (1996) gave participants a battery of cognitive tasks before and after a 60-min bout of cycling at 75% of maximal work capacity and found exercise-induced improvements in tests of executive function and simple reaction time, although performance at choice reaction time and finger tapping tasks was unaffected. Tomporowski et al. (2007) tested participants cycling at 60% of their VO_2_max for up to 120 min with and without fluid replacement and found that executive processing speed improved irrespective of the level of dehydration, although this was accompanied by an increase in errors. In contrast, Moore et al. (2012) reported that participants who cycled at 90% of their ventilatory threshold for 60 min performed worse at a complex perceptual discrimination task than participants who rested for an equivalent amount of time. Detection speed in a memory demanding vigilance task was also increased in participants who exercised, although detection sensitivity did not suffer. Thus, when considered together the effects of exercise-induced fatigue on cognitive performance in general are unclear, and the specific effects on selective attention and cognitive control remain untested.

The aim of the present study was to test performance on a selective attention task at several stages throughout an extended bout of steady state exercise. Participants cycled for 2 h and 16 min in total, stopping every 17 min to perform a selective attention task. A control group also completed the task the same number of times as the exercise group, but without the exercise. The behavioral task was a hybrid flanker visual search task designed to measure overall search performance as a function of task difficulty and the distraction caused by task-irrelevant stimuli that mapped onto competing responses (e.g., Lavie and Cox, 1997; Lavie and Fox, 2000; Forster and Lavie, 2007). The design of this task meant that visual search difficulty and distractor interference could be manipulated independently, thus we were able to obtain indices of both overall search performance and selectivity.

Based on existing evidence from studies using protocols with brief, acute bouts of exercise (McMorris and Graydon, 1997; Aks, 1998) we predicted overall enhanced visual search performance in the early stages of our study. Previous flanker studies have reported either reduced distraction as a function of an acute bout of exercise (Davranche et al., 2009) or no effects (Hillman et al., 2003; Themanson and Hillman, 2006), so it is possible that exercise may either enhance or have no effect on distractibility in the early stages of activity. Conversely, at later stages of the testing session, there may be a decline in performance as participants become increasingly fatigued (Moore et al., 2012). Furthermore, given that several studies have demonstrated superior performance in high-fit individuals compared with low-fit individuals (Colcombe et al., 2004; Themanson and Hillman, 2006), we predicted there may also be a relationship between fitness level and aspects of performance at this task.

## MATERIALS AND METHODS

### PARTICIPANTS

Twenty-eight adult volunteers (14 exercise group, 14 control group) who were students at the University of California, Santa Barbara, took part in the study, either in exchange for course credit or for financial compensation of $10 per hour. The sample size was determined based on similar studies in the cognition/exercise literature (Themanson and Hillman, 2006; Moore et al., 2012) and previous studies from our lab that have used manipulations of task load and response competition (Giesbrecht et al., 2007; Sy et al., 2013, 2014). All participants read and signed a consent form at the beginning of the session. All procedures were approved by the UCSB Human Subjects Committee and the US Army Human Research Protection Office.

One male was removed from the exercise group as he became exceptionally tired midway through the study and was unable to maintain the required workload. An additional male was excluded from the control group due to a failure of the heart rate monitor. Demographic and fitness data from the remaining 26 participants are reported in **Table 1**, along with independent samples *t*-tests confirming no significant group differences. All participants completed the physical activity readiness questionnaire (PAR-Q; National Academy of Sports Medicine, USA) in order to determine their eligibility to participate in aerobic activity. All participants reported having normal or corrected to normal vision.

**Table 1:** Mean and SD values for demographic and cardiovascular data.

Measure	Exercise group	Control group	*t*-test	*p*-value
*n*	13 (6 males)	13 (6 males)	–	–
Age (years)	20.15 (0.69)	20.61 (1.19)	$-$1.21	0.24
Height (cm)	164.97 (5.02)	165.85 (7.85)	$-$0.34	0.74
Weight (kgs)	58.91 (6.18)	61.69 (8.29)	-0.94	0.36
BMI (kg/m^2^)	21.73 (2.93)	22.44 (3.00)	-0.61	0.87
Resting heart rate (BPM)	68.85 (7.90)	70.91 (7.90)	-0.67	0.51
VO_2_max (ml/kg/min)	45.85 (8.86)	41.51 (10.46)	1.29	0.21

### VISUAL SEARCH TASK

**FIGURE 1:**
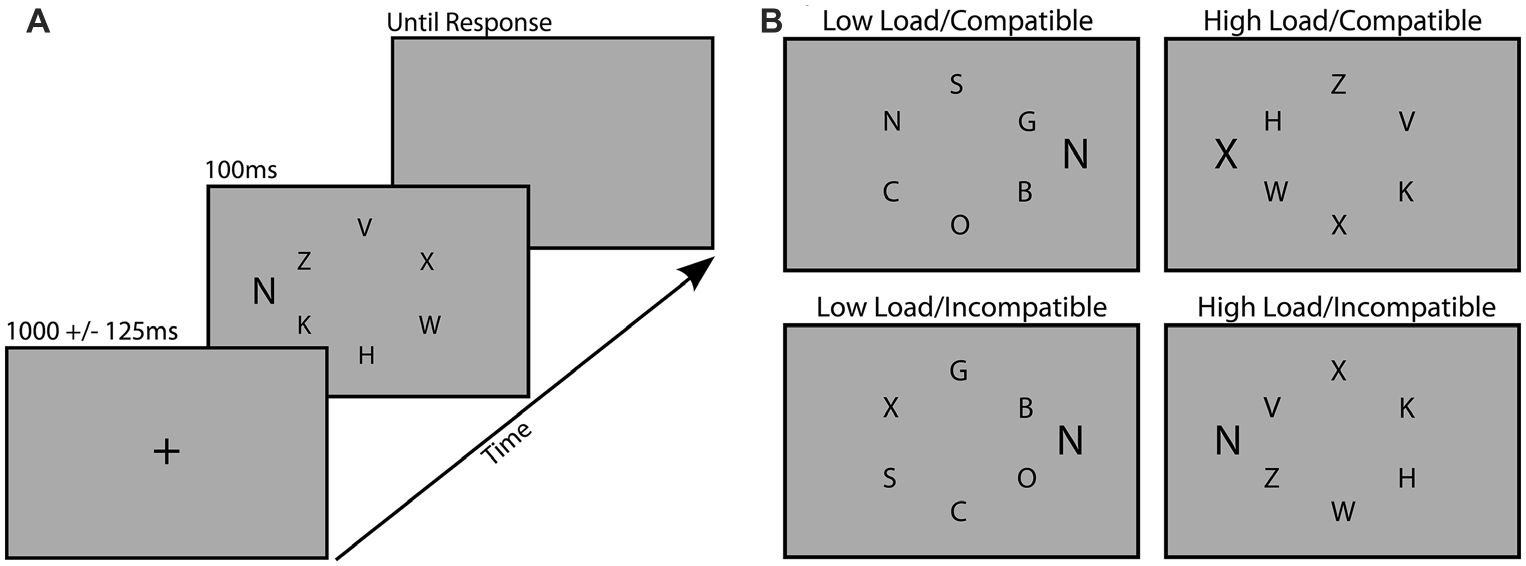
**Examples of the trial procedure (A) and the four different load/distraction conditions (B)**.

The task was designed to measure distraction during visual search, and was based closely on a task developed by Lavie and Cox (1997). All stimuli were presented on an 18″ monitor with custom scripts that utilized the Psychophysics Toolbox for MATLAB (Brainard, 1997). Participants viewed the screen at a distance of 57 cm. Each trial of the search task consisted of a centrally presented fixation cross (1000 ms ± 125 ms), followed by the search array (100 ms) and then a blank gray screen (31.2 cd/m^2^) which remained on screen until a response was made (**Figure 1A**). Each search array consisted of six black upper-case letters (12.5 cd/m^2^) subtending 0.6° by 0.4°, arranged in a circle subtending 2° from a central fixation point. Participants were instructed to search for a target letter (X or N) among an array of non-target letters and respond by pressing the corresponding key on the keyboard as rapidly and accurately as possible. Task difficulty was manipulated between blocks by requiring participants to search for the target among dissimilarly shaped, curvy letters (SCOGB) in the low load condition, and similarly shaped, angular letters (HKVWZ) in the high load condition. Distraction was also manipulated by presenting a task-irrelevant flanker letter (0.8° by 0.5°) to the right or left of the search array, 1.4° from the nearest non-target letter (**Figure 1B**). This flanker was either compatible (same letter) or incompatible (different letter) with the distractor. Target and non-target positions were randomized across trials and the distractor was equally likely to appear on the left or right of the search array.

### SUBMAXIMAL VO_2_max TESTING AND EXERCISE INTENSITY CALCULATION

A measure of estimated maximal oxygen consumption (VO_2_max) was obtained from each subject by having them mount a stationary bike (CycleOps 400 Pro, Saris Cycling Group, WI, USA) and complete the Astrand-Ryming Submaximal Bike Test (Åstrand and Ryhming, 1954). The test involved a 5-min warm-up at a low pedaling resistance producing ∼40 Watts (W) of power, followed by a 6-min test phase at a higher pedaling resistance producing between 80 and 150W, depending on individual fitness, followed by a 2-min cool-down at 40 W. The goal was to elevate the subject’s heart rate to a relatively stable level above 120 BPM in the final 2-min of the test phase. Heart rate was recorded at minutes five and six of the test phase using a CycleOps wireless heart rate monitor, and the mean of these two values, along with the subject’s power output, were used to calculate an estimate of absolute VO_2_max (mL⋅min^-1^) in accordance with the guidelines in Åstrand and Ryhming (1954). An estimate for relative VO_2_max (mL⋅kg^-1^⋅min^-1^) was then calculated by dividing the value for absolute VO_2_max by the subject’s body mass (kg), in accordance with ACSM guidelines (ACSM, 2007, p. 7).

The goal was to have participants work at ∼50% of their VO_2_max, so an individual working VO_2_ value was calculated for each subject by dividing their relative VO_2_max value by two (ACSM, 2007, p. 29). This VO_2_ value and the subject’s body mass were then used in the ACSM leg ergometer equation (Equation 1; ACSM, 2007, p. 47) to calculate an appropriate, approximate pedaling resistance level (kgm/min). This value was converted to Watts and used throughout the study.

Equation 1: ACSM leg ergometer equation (ACSM, 2007, p. 47)

$${VO}_{2}\,\;\;mL\cdot {kg}^{-1}\cdot \min^{-1}=1.8\frac{work\,\;rate\,\;\left(kg\cdot m/\min\right)}{bodymass\left(kg\right)}+7$$

### PROCEDURE

#### Exercise and test sessions

Participants were briefed on the nature of the experiment and the duration and intensity of exercise that they would be undertaking. After signing a consent form, participants were randomly assigned to either the exercise or control group. All participants arrived with the expectation that they would be engaging in exercise; this was necessary to ensure no differences in exercise anticipation anxiety between the groups. Participants were fitted with a heart rate monitor and then sat still and relaxed while baseline heart rate activity was recorded.

Both groups then completed practice trials of the task followed by one session of the visual search task (64 trials low load, 64 trials high load) to establish baseline search task performance. Participants assigned to the exercise group completed the Astrand-Rhyming submaximal bike test, then remounted the bike and cycled for 15 min at ∼50% of their VO_2_max (mean resistance level = 91 W, SD = 24 W), followed by a 2 min cool-down with minimal resistance (40 W). They then dismounted the bike, sat on a chair in front of a computer and completed one session of the visual search task. This exercise > cool-down > search task procedure was then repeated seven more times, so that in total each subject completed the search task nine times interspersed with eight exercise sessions. In total, exercise participants pedaled for 2 h total at ∼50% of VO_2_max, and 16 min at 40 w (cooling down at the end of each session). Participants assigned to the control group completed the same number of sessions at the same times as the exercise group, but rather than exercising for 15 + 2 min between sessions they just sat quietly doing nothing. Control participants were given the Astrand-Ryming test at the end of the session.

#### Ratings of perceived exertion

At the start of the study participants were familiarized with the Ratings of perceived exertion (RPE) scale (Borg, 1970, 1982). Exercise group participants verbally reported ratings of perceived exertion three times during each exercise block (at 4, 9, and 14 min). RPE is a subjective rating of the intensity of physical sensations a person experiences during physical activity, including increased heart rate, respiration rate, muscle fatigue and physical discomfort. When prompted by the experimenter, participants appraised their feeling of exertion by viewing a scale and reporting a number between six (no exertion) and 20 (maximal exertion).

#### Saliva samples

Saliva samples were collected from both groups prior to the baseline task block, and then after task sessions two, four, six, and eight. The aim was to obtain measures of salivary cortisol and alpha amylase as participants progressed through the testing session. Bouts of physical activity are associated with increases in salivary cortisol (e.g., Chicharro et al., 1998) and alpha-amylase (Li and Gleeson, 2004), so these measures would help confirm that our stress manipulation was effective. For each saliva sample, participants passively drooled 2 ml of saliva into a plastic vial via a plastic drinking straw. Samples were immediately frozen for storage at -20°C. To ensure the samples were accurate and high quality, participants were required to not drink within a 10-min period prior to the collection of each saliva sample, as consumption of liquid within this period can dilute the sample. Participants were allowed unrestricted access to water at all other times during the testing session. Consumption of food or any other types of liquid was not permitted. Samples were shipped on dry ice for analysis at the Clinical Endocrinology Laboratory, UC Davis, Davis, CA, USA.

### DESIGN

Three within-participants variables were manipulated. First, visual search performance was sampled nine times throughout the testing session. Second, distractibility was measured by manipulating whether the task-irrelevant letter presented outside of the search array was compatible or incompatible with the target. Comparing speed and accuracy between the different distraction conditions provided an index of distractor interference. Third, search task difficulty was manipulated by presenting non-target items in the search array that were either similarly shaped (high load) or dissimilarly shaped (low load) to the target. A number of studies have demonstrated that distraction is typically more robust under conditions of low visual search load (e.g., Lavie and Cox, 1997; Forster and Lavie, 2007).

### ANALYSES

Data from 26 participants (13 exercise, 13 control) were collapsed from nine sessions (baseline, 17, 34, 51, 68, 85, 102, 119, 136 min) into five sessions (baseline, 34, 68, 102, 136 min) to increase statistical power. The 34, 68, 102, and 136 min sessions will be collectively referred to as the “test” sessions from here onward. *Post hoc* analyses for main effects and interactions of interest were computed using paired or independent samples *t*-tests correcting for multiple comparisons using the false discovery rate method with a threshold of 0.05 (Benjamini and Hochberg, 1995). For completeness, both the uncorrected and FDR adjusted *p*-values (*q*) are reported for the *post hoc* tests.

## RESULTS

The results are reported in three sections. First, we evaluate the physiological data to confirm the effectiveness of the physical fatigue manipulation. Second, we analyze the behavioral data to determine whether exercise had any impact on search task performance. Third, we examine the relationship between individual aerobic capacity (VO_2_max) and search task performance.

### PHYSIOLOGICAL DATA

#### Ratings of perceived exertion

**FIGURE 2:**
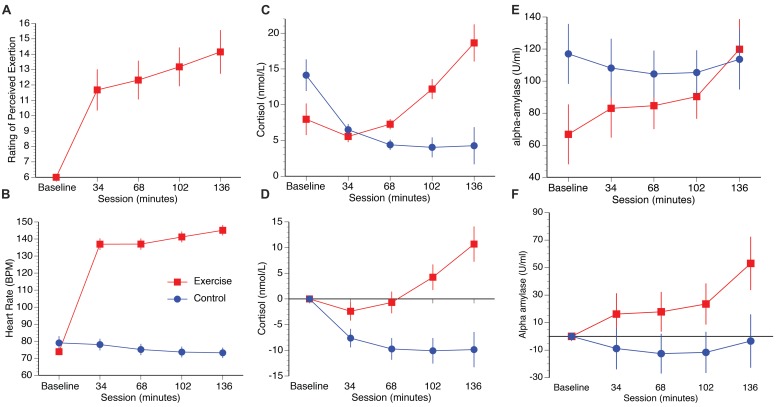
**Plots illustrate RPE (A), heart rate (B), raw cortisol and alpha-amylase data (C,E), and cortisol and alpha-amylase data corrected to baseline (D,F)**. Error bars represent SEM.

Participants in the exercise group perceived their exertion to be “light/somewhat hard” in the first half of the session, increasing to “somewhat hard” after 102 min and “somewhat hard/heavy” at 136 min (**Figure 2A**). A repeated measures ANOVA on the exercise group (the baseline condition was excluded because participants were not exercising at this stage) with the within participants’ factor session (34, 68, 102, 136 min) confirmed a significant increase in mean RPE as a function of time spent cycling [*F*(3,36) = 31.09, *p* < 0.001, η^2^ = 0.72]. *Post hoc* paired samples *t*-tests confirmed significant increases between 34 and 68 min [*t*(12) = -2.79, *p* = 0.016, *q* = 0.016], 68 and 102 min [*t*(12) = -4.39, *p* = 0.001, *q* = 0.002], and 102 to 136 min [*t*(12) = -7.45, *p* = 0.001, *q* = 0.002].

#### Heart rate

Heart rate increased in the exercise group as a function of time spent cycling, and decreased slightly over the course of the experiment in the control group (**Figure 2B**). A repeated measures ANOVA with session as the within participants factor (34, 68, 102, 136 min) and group as the between participants factor (exercise, control) confirmed significant main effects of session [*F*(4,96) = 196.62, *p* = 0.008, η^2^ = 0.89], group, [*F*(1,24) = 166.98, *p* < 0.001, η^2^ = 0.72] and a session × group interaction [*F*(4,96) = 308.60, *p* < 0.001, η^2^ = 0.93]. Within-group direct comparisons confirmed significant increases in heart rate in the exercise group from baseline to 34 min [*t*(12) = 19.13, *p* < 0.001, *q* = 0.004], 68 to 102 mins [*t*(12) = 4.70, *p* = 0.001, *q* = 0.004], and from 102 to 136 mins [*t*(12) = 3.16, *p* = 0.008, *q* = 0.016]. In contrast, heart rate decreased slightly over the course of the session in the control group, with a significant drop from 34 to 68 min [*t*(12) = 3.26, *p* = 0.007, *q* = 0.016].

#### Salivary cortisol and alpha-amylase

Salivary cortisol levels increased in the exercise group relative to the control group over the duration of the test session. Raw and baseline corrected cortisol data are presented in **Figures 2C,D**, respectively. A mixed measures ANOVA with session (34 m, 68 m, 102 m, 136 m] as the within participants factor, and group (exercise, control) as the between participants factor, was computed for the baseline corrected salivary cortisol data. The analysis revealed main effects of session [*F*(3,72) = 7.84, *p* < 0.001, η^2^ = 0.25], group [*F*(1,24) = 15.59, *p* = 0.001, η^2^ = 0.39], and a session by group interaction, [*F*(3,72) = 12.73, *p* < 0.001, η^2^ = 0.34]. Direct comparisons between the exercise and control groups confirmed that cortisol was significantly higher in the exercise group than the control group at 34 min [*t*(24) = 2.11, *p* = 0.046, *q* = 0.046], 68 min [*t*(24) = 3.11, *p* = 0.01, *q* = 0.007], 102 min [*t*(24) = 4.17, *p* < 0.001, *q* = 0.002], and 136 min [*t*(24) = 4.31, *p* < 0.001, *q* = 0.002]. Raw and baseline corrected alpha-amylase data are presented in **Figures 2E,F**, respectively. Although the alpha-amylase data appear to follow a pattern that is similar to the cortisol data, a mixed measures ANOVA computed for the baseline corrected data with the same factors reported previously found no main effect of session [*F*(3,72) = 1.99, *p* = 0.12, η^2^ = 0.08], group [*F*(1,24) = 3.98, *p* = 0.057, η^2^ = 0.14], or interaction of session by group [*F*(3,72) = 0.86, *p* = 0.46, η^2^ = 0.04].

### BEHAVIORAL DATA

#### Reaction time

**FIGURE 3:**
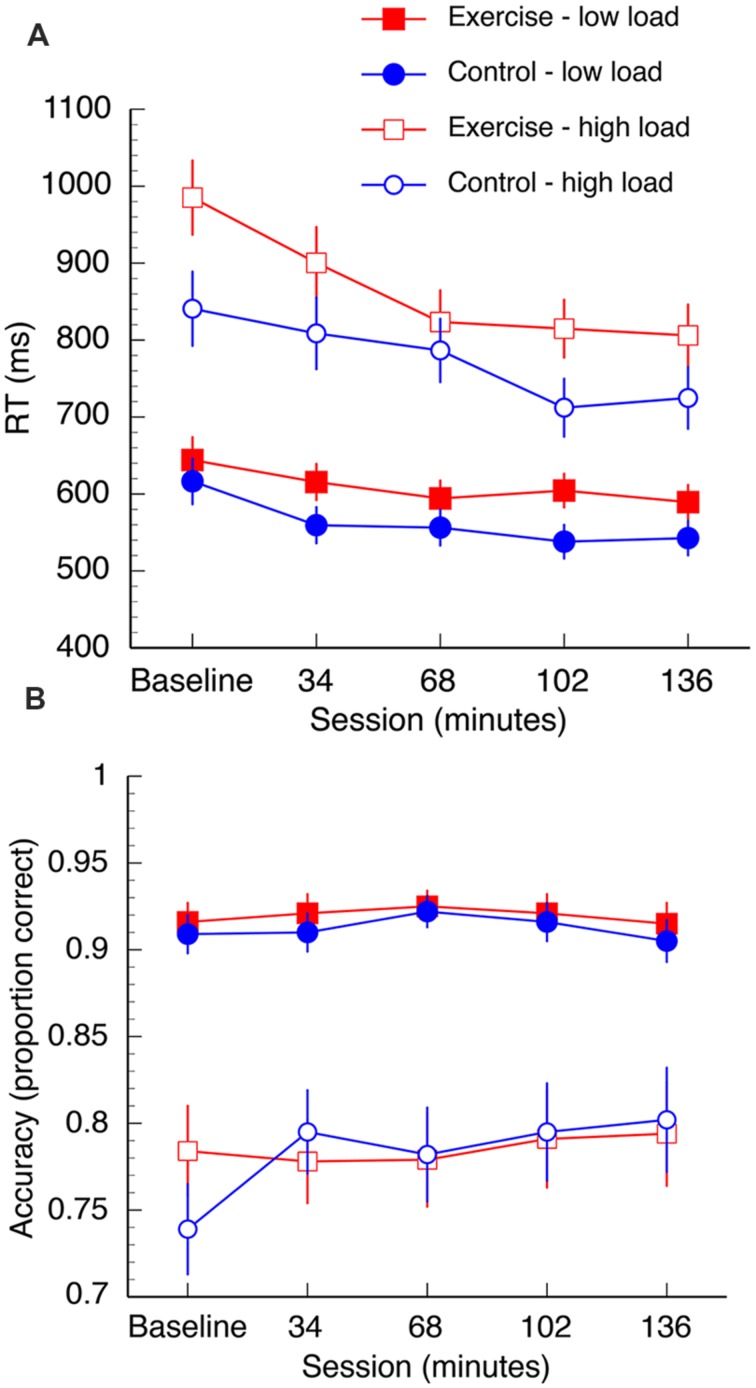
**Mean reaction time (A) and accuracy data (B) for both groups.** Data are collapsed over “distractibility” as this variable was not found to interact with any other variables. Error bars represent SEM.

**FIGURE 4:**
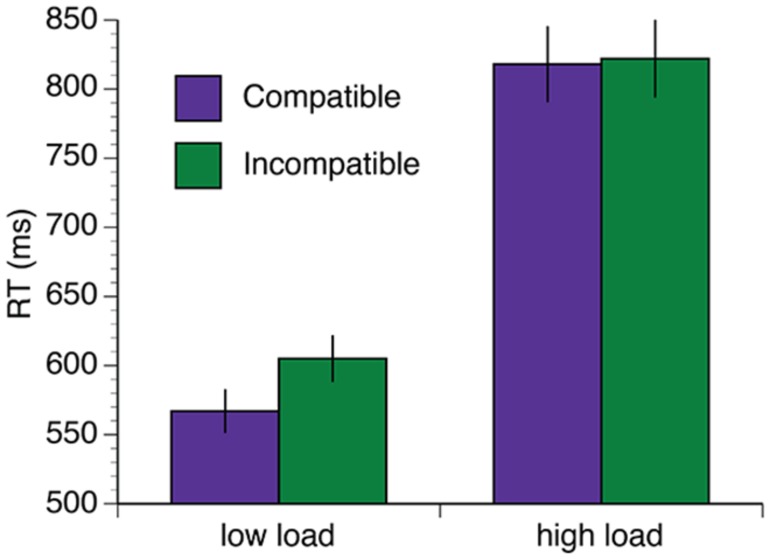
**Distractor incompatibility effect under low load but not high load.** Error bars represent SEM.

The mean RT data are shown in **Figure 3A**. A 2 (group: exercise, control) × 2 (load: low, high) × 2 (interference: compatible, incompatible) × 5 (session: baseline, 34, 68, 102, 136 min) repeated measures ANOVA was computed for the mean RT data. Overall, there was a reduction in RT in both groups over the duration of the experiment [*F*(4,96) = 19.61, *p* < 0.001, η^2^ = 0.45]. There was also a significant session × load interaction [*F*(4,96) = 8.46, *p* < 0.001, η^2^ = 0.26]. This interaction was driven by significant reductions in RT under high task load between 34and 68 min [*t*(25) = 3.04, *p* = 0.006, *q* = 0.016] and 68–102 min [*t*(25) = 3.16, *p* = 0.004, *q* = 0.016], and under low task load between baseline – 34 min [*t*(25) = 4.57, *p* < 0.002, *q* = 0.016]. Both groups also demonstrated increased RT under conditions of high load [*F*(1,24) = 214.35, *p* < 0.001, η^2^ = 0.90] and longer RTs when the distractors were incompatible distractors [*F*(1,24) = 27.91, *p* < 0.001, η^2^ = 0.54]. Furthermore, there was a load × interference interaction [*F*(1,24) = 26.00, *p* < 0.001, η^2^ = 0.52]. *Post hoc* paired samples *t*-tests confirmed that incompatible distractors significantly increased RTs under low load, [*t*(26) = -10.69, *p* < 0.001, *q* = 0.002], but not high load [*t*(26) = 0.35, *p* = 0.73, *q* = 0.73; **Figure 4**].

There was also a significant three way interaction between group, session, and load on search task performance [*F*(4,96) = 2.49, *p* = 0.048, η^2^ = 0.09]. In the first half of the session both groups appear to demonstrate a learning trend as they complete more blocks of the task, although *post hoc* paired samples *t*-tests confirm that the only statistically significant decrease in RT is from baseline to 34 min in the control group under low load [*t*(12) = 4.33, *p* = 0.001, *q* = 0.008]. At 68 min, significant differences emerge between the two groups under high task load: there is a significant decrease in RT from 68 to 102 min [*t*(12) = 3.93, *p* = 0.002, *q* = 0.008] in the control group, but no change in the exercise group [*t*(12) = 0.64, *p* = 0.53, *q* = 0.53]. RT did not significantly change between 102 and 136 min under high task load in either the exercise group [*t*(12) = 0.66, *p* = 0.52, *q* = 0.53] or control group [*t*(12) = -1.02, *p* = 0.35, *q* = 0.55]. The groups did not differ at baseline when corrected for multiple comparisons [*t*(24) = 2.1, *p* = 0.04, *q* = 0.53).

#### Accuracy

The mean accuracy data are shown in **Figure 3B**. A mixed measures ANOVA (same factors as listed in the previous section) was computed for the accuracy data. There was an overall decline in accuracy in both groups under high load [*F*(1,24) = 108.88, *p* < 0.001, η^2^ = .82] and high interference [*F*(1,24) = 26.00, *p* < 0.001, η^2^ = 0.52]. Accuracy improved in both groups over the duration of the experiment [*F*(4,96) = 2.74, *p* = 0.03, η^2^ = 0.10]. There was a session by load interaction [*F*(4, 96) = 3.05, *p* = 0.02, η^2^ = 0.11]. Follow up analyses revealed marginally improved performance between performance at baseline and after 34 min in the high load condition, but this test did not survive correction for multiple comparisons [*t*(25) = -2.15, *p* = 0.041, *q* = 0.32]. There were no overall group differences [*F*(1,24) = 0.04, *p* = 0.84, η^2^ = 0.02].

### CARDIOVASCULAR FITNESS AND BEHAVIORAL PERFORMANCE

**FIGURE 5:**
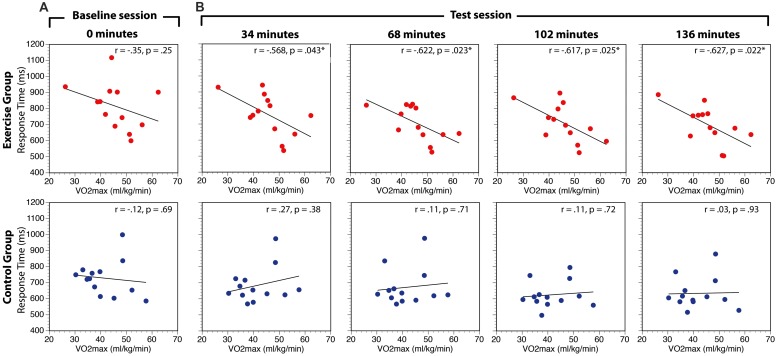
**Correlations between VO_2_max and visual search RT at baseline (A) and during the test session (B).** RT data are averaged across search task load and distractor interference conditions. While the *p*-values indicate the values uncorrected for multiple comparisons, *indicate the hypothesis tests that are *p* < 0.05 FDR-corrected for multiple comparisons.

The relationship between aerobic fitness (VO_2_max) and search performance (RT) was assessed in two ways. First, to globally assess this relationship we computed the correlation between VO_2_max and RT in the baseline session and the overall average of the test sessions for experimental and control groups. There was no relationship between VO_2_max and RT in the baseline condition for either the exercise group [*r*(11) = -0.35, *p* = 0.24] or the control group [*r*(11) = -0.12, *p* = 0.69; **Figure 5A**). In contrast, there was a significant negative relationship between VO_2_max and RT averaged across all conditions of the test session for the exercise group, [*r*(11) = -0.63, *p* = 0.02], but not the control group, [*r*(11) = 0.14, *p* = 0.66]. Direct comparisons of the correlation coefficients in the experimental and control conditions using Fisher’s *r*-to-*z* transformations revealed that the correlations were not significantly different in the baseline period (*Z* = -0.55, *p* = 0.59), but were different in the test sessions (*Z* = -1.97, *p* = 0.049). This finding suggests that participants with higher aerobic capacity are faster to respond to search task targets than participants with lower capacity.

Second, to investigate whether the correlation between aerobic fitness and search performance changed over the course of the experiment, separate correlations were calculated within each group at 34, 68, 102, and 136 min correcting for multiple comparisons (*p* < 0.05) within group using the false discovery rate approach (Benjamini and Hochberg, 1995). The results of this analysis are summarized in **Table 2** and **Figure 5B**. There were no significant correlations in the control group at any point during the experiment. In the experimental group, the correlation was significant in all the test sessions.

**Table 2:** Pearson correlations show relationship between VO_2_max and RT for exercise and control groups.

	Baseline	34 min	68 min	102 min	136 min
**Exercise group**
Pearson	-0.35	-0.568	-0.622	-0.617	-0.627
Uncorrected *p*-value	0.25	0.043*	0.023*	0.025*	0.022*
**Control group**
Pearson	-0.12	0.27	0.11	0.11	0.03
Uncorrected *p*-value	0.69	0.38	0.71	0.72	0.93

*indicates *p* < 0.05 FDR-corrected for multiple comparisons.

**FIGURE 6:**
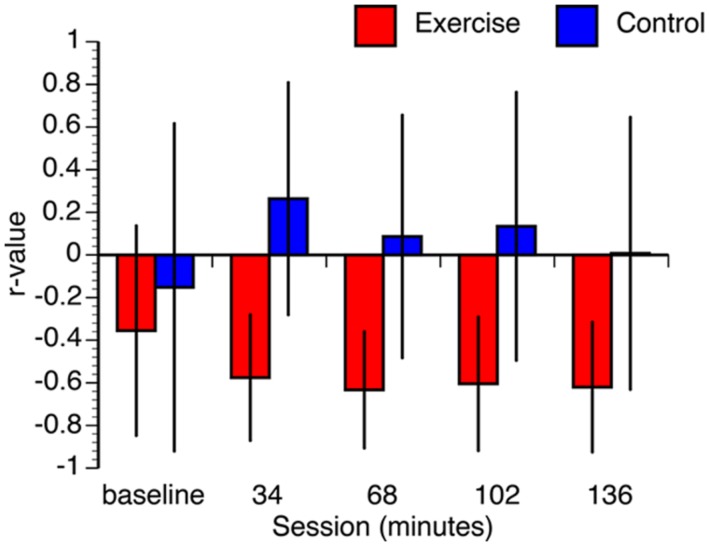
**Bootstrap estimated mean correlation values.** Error bars represent 95% false coverage rate (FCR) confidence intervals that complement the *p* < 0.05 FDR-corrected hypothesis tests.

Given the relatively small sample size, several steps were taken to ensure that the results were not contaminated by deviations in normality and/or differences in baseline measurements. First, we constructed confidence intervals for each correlation using bootstrap resampling with 1000 iterations. To maintain consistency with the FDR-corrected hypothesis tests, we constructed the confidence intervals using the false coverage rate (FCR) procedure (i.e., 95% FCR intervals; Benjamini and Yekutieli, 2005). The mean correlation values of the resampled distributions and the confidence intervals are shown in **Figure 6**. Not only were the means of the bootstrapped correlation coefficients very similar to the actual coefficients, but the bootstrapped confidence intervals corroborated the hypothesis tests (i.e., intervals for the tests that were statistically significant did not include zero). While it is still possible that the observed pattern of correlations may change with a larger sample size, the bootstrap analysis confirms that the results are stable under resampling. Second, we confirmed that all data from both groups are within three standard deviations of the condition means. Third, Kolmogorov-Smirnov tests revealed that the majority of the conditions were normally distributed. The only non-normal data were the 68, 102, and 136 min conditions in the control group. As a result, we also conducted non-parametric Spearman correlations. The results of these non-parametric hypothesis tests matched the parametric tests in every condition. Finally, it is possible that the observed differences in the correlations between VO_2_max and performance between the groups could be associated with differences in the baseline condition. To address this we regressed VO_2_max against baseline RT and overall test RT, for each group independently. In the exercise group, overall test RT significantly predicted VO_2_max, [β = -0.76, *t*(12) = -2.23, *p* = 0.050], whereas baseline RT did not significantly predict VO_2_max, (β = 0.19, *t*(12) = 0.56, *p* = 0.59). The overall model explained a marginally significant proportion of variance in VO_2_max, [*R*^2^ = 0.41, *F*(2,10) = 3.52, *p* < 0.07]. In the control group, overall test RT did not predict VO_2_max, [β = 0.41, *t*(12) = 1.02, *p* = 0.33] and neither did baseline RT, [β = -0.41, *t*(12) = -0.99, *p* = 0.34]. The overall model did not explain the variance in VO_2_max, [*F*(2,10) = 0.60, *p* = 0.57]. These analyses confirm that the observed differences in correlations between VO_2_max and RT between the two groups are not just the result of differences at baseline.

## DISCUSSION

The goal of the present study was to investigate changes in multiple components of visual attention during a long bout of physical activity. Two key findings emerged from the behavioral data. First, the three-way interaction between load, session and group suggests that a long bout of exercise may impact the learning effect in a visual search task. Second, the significant negative relationship that emerges between VO_2_max and search task response time in the exercise group, but not in the control group suggests that aerobic capacity may only be a good predictor of visual search performance in the current task when one is engaged in exercise. In the following sections, we discuss the implications of these findings for both the cognition and exercise literature and for models of selective attention.

### EXERCISE EFFECTS ON SEARCH TASK PERFORMANCE

Between the baseline condition and 68 min, search task RTs trended downward in both groups. However, while the control group showed significant improvement from 68 to 102 min in the high load condition, the exercise group did not. This pattern of results suggests that an hour-long bout of exercise can have a detrimental impact on normal task learning during visual search, but only under demanding task load conditions. One possible explanation is that the acute bout of exercise drains processing resources and while there are still sufficient resources for the learning effect to continue under low search task load, there are not sufficient resources to support learning under high load. There is other similar evidence in the literature suggesting that participants who engage in 1-h of intense exercise are impaired at perceptual discrimination relative to participants that rest for an equivalent amount of time (Moore et al., 2012). Our findings not only provide further evidence for impaired perceptual discrimination after a long bout of acute exercise, but also suggest that task load may have an important role in determining the extent of the impairment. The present result, however, must be interpreted with some caution because of the difference between the groups in baseline performance in the high load condition. Also, although our physical stress manipulation was effective, as confirmed by increased salivary cortisol levels in the exercise group compared to the control group during the test session and increased RPEs over the course of the session in the exercise group, it is possible that the results of the present study may have been more robust if the exercise group had also been required to exercise more intensively. For example, (Moore et al., 2012) required subjects to exercise at a far higher intensity (90% of ventilatory threshold) than our subjects. Given that overall effects on cognitive performance generally tend to be small and affected by a range of behavioral and exercise intensity/duration related factors (e.g., Lambourne et al., 2010; Chang et al., 2012), it is perhaps not surprising that our effects are also small.

### RELATIONSHIP BETWEEN AEROBIC CAPACITY AND SEARCH RT

A robust, significant negative relationship between VO_2_max and RT emerged in the exercise group as soon as they began to exercise, and this relationship endured for the remainder of the session. This relationship was not present in either group during the baseline session, and did not emerge in the control group at any stage of the test session, suggesting that aerobic capacity is related to search performance in this task, but only during a bout of physical exercise. There was no relationship between aerobic capacity and accuracy, indicating that enhancement in processing speed did not come at the cost of increased errors.

To an extent, the discovery that people with higher aerobic capacity outperform people with lower capacity in our search task corroborates previous data that show enhanced cognitive performance in higher-fit people. Themanson and Hillman (2006) monitored brain activity using the event related potential (ERP) technique while subjects performed a flanker task before and after a 30-min bout of treadmill exercise. Although the authors found no effects of the acute bout of exercise on any dependent measures, they did find that higher-fit adults showed reduced error related negativity (ERN), increased error positivity (Pe), and increased post-error response slowing, all of which suggest increased involvement of top–down cognitive control mechanisms.

One may also draw associations between the present findings and results from studies of cognitive vitality in older adults. A meta-analytical study conducted to examine the effects of aerobic fitness training interventions on cognitive performance in sedentary older adults (aged 55–80 years) found that training had robust, reliable effects on cognitive performance across various domains, including executive function and visuospatial task performance (Colcombe and Kramer, 2003). Another study of older adults found that high-fit, aerobically trained participants showed reduced interference effects in an Eriksen Flanker task when compared to low-fit participants (Colcombe et al., 2004). However, our findings are unique in showing that under the present experimental conditions, aerobic capacity may be a relevant factor for visual search proficiency *after* an individual has began an acute bout of exercise. This is in contrast to the vast majority of other research, which demonstrates a relationship between aerobic capacity and cognitive performance while participants are at rest. Thus, there may be a common mechanism whereby aerobic capacity is relevant for certain types of cognitive performance while resting and especially important for other types of cognitive task during an acute bout of exercise.

It is possible that common mechanisms can account for these results and evidence for this possibility comes from both animal and human studies. Animal models demonstrate that increased aerobic fitness is associated with higher levels of BDNF, a growth factor that protects and supports the function and survival of neurons (Neeper et al., 1995). This, and other neurotrophins are responsible for the survival of neurons (Lewin and Barde, 1996), the regulation of neuronal connectivity and synaptic efficacy (Lu and Chow, 1999) and neurogenesis (van Praag et al., 1999). Thus, aerobic activity results in greater neural efficiency and plasticity, meaning that animals with greater aerobic capacity can show improved cognitive performance (van Praag et al., 1999). There is also evidence that BDNF plays a key role in the human brain. Knaepen et al. (2010) carried out a systematic review of studies that evaluated the effects of acute exercise or a training intervention on human participants. They conclude that aerobic exercise can result in both higher BDNF synthesis and upregulation of the synthesis, reabsorption and release of BDNF by cells, thus inducing both neuroprotective and neurotrophic effects. It is therefore possible that increased cardiovascular fitness leads to an increased number of synapses in frontal and parietal regions of the human brain (Colcombe et al., 2004). It may also be the case that enhancements in oxygen transportation to the brain that are associated with higher levels of cardiovascular fitness (Endres et al., 2003; Swain et al., 2003) may also boost cognitive performance due to the increased availability of metabolites to neurons (Chodzko-Zajko and Moore, 1994). The physiological changes associated with these gains in aerobic fitness have been referred to as the cardiovascular fitness hypothesis (North et al., 1990; Etnier et al., 2006). Critically, within the present context it is plausible that this increased interconnectivity and metabolic efficiency may allow for greater recruitment and supply of neurons under conditions in which metabolic demands are increased. This may explain why higher-fit participants in our study were able to search for the target more efficiently than low-fit participants during the prolonged bout of exercise.

### IMPLICATIONS FOR MODELS OF SELECTIVE ATTENTION

#### Neural theory of visual attention

In addition to providing insight onto the complex interaction between physical fatigue and cognition, the present results can be interpreted within the context of models of attention that are not explicitly designed to explain these effects. Consider the theory of visual attention (TVA: Bundesen, 1990) – a computation theory that is able to account for a wide range of aspects of the operation of selective attention. TVA is based on two equations, which, together, account for two fundamental aspects of selective attention: filtering (object selection) and pigeonholing (feature selection). The updated neural TVA (NTVA; Bundesen et al., 2005) is an attempt to account for these mechanisms at the neural level. According to the model, filtering changes the number of cortical neurons that represent an object, whereas pigeonholing changes activation levels in neurons responsible for coding particular features. Together, these two mechanisms are responsible for controlling the activity levels of populations of neurons responsible for signaling specific object categories. These populations then compete with populations of neurons in the visual system responsible for signaling other object categories, with the level of activation of each population determining whether a category will be encoded into visual short-term memory. According to NTVA, if a behaviorally relevant object has a high attentional weight then a larger set of neurons representing that object should be active. Having large populations of neurons available to represent the behaviorally relevant object is therefore vital for the rapid and accurate categorization of that object. Within the present context, if high aerobic capacity translates into increased synaptic interconnectivity and metabolic supply allowing for the recruitment of greater numbers of neurons, then individuals with higher aerobic capacity may be more readily able to recruit larger populations of neurons to represent the behaviorally relevant object, and readily supply those neurons with the necessary metabolites. Hence aerobic fitness may become an important factor in determining speed of behaviorally relevant object identification under conditions of high metabolic load, such as during or immediately after an acute bout of exercise.

#### Perceptual load theory

Conditions of high perceptual load lead to reduced interference from competing distractors (Lavie and Tsal, 1994; Lavie, 1995; see Lavie, 2006, 2010, for reviews), supporting the idea that attention is a limited capacity resource. Although perceptual load theory does not make any specific predictions regarding the effects of psychological or physiological stress and fatigue on selectivity, Lavie and Tsal (1994) acknowledge that in addition to perceptual load, attentional capacity can also be modulated by factors such as the temporal state of alertness and availability of resources. For example, evidence from hybrid flanker visual search tasks similar to the one used in the present study indicates that mental fatigue leads to increased distractor interference under low perceptual load (Csathó et al., 2012), and social stress causes reduced distractor interference under low load and increased interference under high load (Sato et al., 2012). Although the presence of a significant load × distraction interaction in our data confirmed that distractor interference was greater under low load than high load, this interference effect was not modulated by exercise at any point during the test session. This result concurs with previous studies that demonstrate an acute bout of exercise does not interact with flanker interference in an Eriksen flankers task (Hillman et al., 2003; Themanson and Hillman, 2006; Davranche et al., 2009). However, it appears there is a contrast between physical activity induced stress, which does not impact upon distractor interference, and psychological stress and fatigue, which are known to interact with distractor interference in this task (Csathó et al., 2012; Sato et al., 2012).

## CONCLUSION

Relatively few attempts have been made to study the effects of prolonged exercise on cognitive performance in the laboratory, and the effects of exercise-induced arousal and fatigue on selective attention and cognitive control are unclear. In the present study, the data suggest that aerobic capacity may be an important determinant of visual search performance, with high-fit participants able to identify a target more rapidly than low-fit participants during a bout of physical exercise. We also provide tentative evidence that prolonged exercise can have a detrimental effect on visual search under high search task load.

Our findings are important and unique for five main reasons. First, these data are the first to suggest that a relationship between aerobic capacity and cognitive performance can emerge after a bout of physical exercise. Second, our study is the first to test exercise effects on visual search and perceptual distraction. Third, the present study is one of a handful of studies that test the effects of a prolonged bout of exercise on cognitive performance. Fourth, while a relationship between aerobic capacity and cognitive performance is well established in aging populations (Colcombe and Kramer, 2003), our study is one of a handful that also demonstrates this effect in a sample of young, healthy adults. Finally, the present results have implications for the generality of current theories of selective attention, which are largely based on behavioral performance measured at rest. While there appears to be a link between the physiological changes that occur with exercise and cognitive function that is consistent with the NTVA, future work is needed to investigate the empirical viability of this link.

## Conflict of Interest Statement

The authors declare that the research was conducted in the absence of any commercial or financial relationships that could be construed as a potential conflict of interest.

## References

[B1] ACSM. (2007). *ACSM’s Metabolic Calculations Handbook*. Baltimore, MD: Lippincott Williams and Wilkins.

[B2] Aks D. J. (1998). Influence of exercise on visual search: implications for mediating cognitive mechanisms. *Percept. Mot. Skills* 87(3Pt 1) 771–783 10.2466/pms.1998.87.3.771 9885036

[B3] Åstrand P.-O. Ryhming I. (1954). A nomogram for calculation of aerobic capacity (physical fitness) from pulse rate during submaximal work. *J. Appl. Physiol.* 7 218–221. 13211501 10.1152/jappl.1954.7.2.218

[B4] Bard C. Fleury M. (1978). Influence of imposed metabolic fatigue on visual capacity components. *Percept. Mot. Skills* 47(3Pt 2), 1283–1287 10.2466/pms.1978.47.3f.1283 745914

[B5] Benjamini Y. Hochberg Y. (1995). Controlling the false discovery rate: a practical and powerful approach to multiple testing. *J. R. Stat. Soc.* 57 289–300 10.2307/2346101

[B6] Benjamini Y. Yekutieli D. (2005). False discovery rate–adjusted multiple confidence intervals for selected parameters. *J. Am. Stat. Assoc.* 100 71–81 10.1198/016214504000001907

[B7] Borg G. (1970). Perceived exertion as an indicator of somatic stress. *Scand. J. Rehabil. Med.* 2 92–98. 5523831

[B8] Borg G. (1982). Psychophysical bases of perceived exertion. *Med. Sci. Sports Exerc.* 14 377–381 10.1249/00005768-198205000-00012 7154893

[B9] Brainard D. H. (1997). The psychophysics toolbox. *Spat. Vis.* 10 433–436 10.1163/156856897X00357 9176952

[B10] Bundesen C. (1990). A theory of visual attention. *Psychol. Rev.* 97 523–547 10.1037/0033-295X.97.4.523 2247540

[B11] Bundesen C. Habekost T. Kyllingsbæk S. (2005). A neural theory of visual attention: bridging cognition and neurophysiology. *Psychol. Rev.* 112 291–328 10.1037/0033-295X.112.2.291 15783288

[B12] Chang Y. K. Labban J. D. Gapin J. I. Etnier J. L. (2012). The effects of acute exercise on cognitive performance: a meta-analysis. *Brain Res.* 1453 87–101 10.1016/j.brainres.2012.02.068 22480735

[B13] Chicharro J. L. Lucía A. Pérez M. Vaquero A. F. Ureña R. (1998). Saliva composition and exercise. *Sports Med.* 26 17–27 10.2165/00007256-199826010-00002 9739538

[B14] Chodzko-Zajko W. J. Moore K. A. (1994). Physical fitness and cognitive functioning in aging. *Exerc. Sport Sci. Rev.* 22 195–220 10.1249/00003677-199401000-00009 7925543

[B15] Colcombe S. Kramer A. F. (2003). Fitness effects on the cognitive function of older adults: a meta-analytic study. *Psychol. Sci.* 14 125–130 10.1111/1467-9280.t01-1-01430 12661673

[B16] Colcombe S. Kramer A. F. Erickson K. I. Scalf P. McAuley E. Cohen N. J. (2004). Cardiovascular fitness, cortical plasticity, and aging. *Proc. Natl. Acad. Sci. U.S.A.* 101 3316–3321 10.1073/pnas.0400266101 14978288 PMC373255

[B17] Csathó Á. van der Linden D. Hernádi I. Buzás P. Kalmár G. (2012). Effects of mental fatigue on the capacity limits of visual attention. *J. Cogn. Psychol.* 24 511–524 10.1080/20445911.2012.658039

[B18] Davranche K. Burle B. Audiffren M. Hasbroucq T. (2005). Information processing during physical exercise: a chronometric and electromyographic study. *Exp. Brain Res.* 165 532–540 10.1007/s00221-005-2331-9 15883799

[B19] Davranche K. Hall B. McMorris T. (2009). Effect of acute exercise on cognitive control required during an Eriksen flanker task. *J. Sport Exerc. Psychol.* 31 628–639. 20016112 10.1123/jsep.31.5.628

[B20] Endres M. Gertz K. Lindauer U. Katchanov J. Schultze J. Schröck H. (2003). Mechanisms of stroke protection by physical activity. *Ann. Neurol.* 54 582–590 10.1002/ana.10722 14595647

[B21] Eriksen B. Eriksen C. (1974). Effects of noise letters upon the identification of a target letter in a nonsearch task. *Percept. Psychophys.* 16 143–149 10.3758/BF03203267

[B22] Etnier J. L. Nowell P. M. Landers D. M. Sibley B. A. (2006). A meta-regression to examine the relationship between aerobic fitness and cognitive performance. *Brain Res. Rev.* 52 119–130 10.1016/j.brainresrev.2006.01.002 16490256

[B23] Ferris L. T. Williams J. S. Shen C.-L. (2007). The effect of acute exercise on serum brain-derived neurotrophic factor levels and cognitive function. *Med. Sci. Sports Exerc.* 39 728–734 10.1249/mss.0b013e31802f04c7 17414812

[B24] Forster S. Lavie N. (2007). High perceptual load makes everybody equal: eliminating individual differences in distractibility with load. *Psychol. Sci.* 18 377–381 10.1111/j.1467-9280.2007.01908.x 17576274

[B25] Giesbrecht B. Sy J. L. Elliott J. C. (2007). Electrophysiological evidence for both perceptual and postperceptual selection during the attentional blink. *J. Cogn. Neurosci.* 19 2005–2018 10.1162/jocn.2007.19.12.2005 17892389

[B26] Hillman C. H. Snook E. M. Jerome G. J. (2003). Acute cardiovascular exercise and executive control function. *Int. J. Psychophysiol.* 48 307–314 10.1016/S0167-8760(03)00080-1 12798990

[B27] Hogervorst E. Riedel W. Jeukendrup A. Jolles J. (1996). Cognitive performance after strenuous physical exercise. *Percept. Mot. Skills* 83 479–488 10.2466/pms.1996.83.2.479 8902021

[B28] Knaepen K. Goekint M. Heyman E. M. Meeusen R. (2010). Neuroplasticity – exercise-induced response of peripheral brain-derived neurotrophic factor: a systematic review of experimental studies in human subjects. *Sports Med.* 40 765–801 10.2165/11534530-000000000-00000 20726622

[B29] Lambourne K. Audiffren M. Tomporowski P. D. (2010). Effects of acute exercise on sensory and executive processing tasks. *Med. Sci. Sports Exerc.* 42 1396–1402 10.1249/MSS.0b013e3181cbee11 20019631

[B30] Lambourne K. Tomporowski P. (2010). The effect of exercise-induced arousal on cognitive task performance: a meta-regression analysis. *Brain Res.* 1341 12–24 10.1016/j.brainres.2010.03.091 20381468

[B31] Lavie N. (1995). Perceptual load as a necessary condition for selective attention. *J. Exp. Psychol.* 21 451–468 10.1037/0096-1523.21.3.451 7790827

[B32] Lavie N. (2006). The role of perceptual load in visual awareness. *Brain Res.* 1080 91–100 10.1016/j.brainres.2005.10.023 16413514

[B33] Lavie N. (2010). Attention, distraction, and cognitive control under load. *Curr. Dir. Psychol. Sci.* 19 143–148 10.1177/0963721410370295

[B34] Lavie N. Cox S. (1997). On the efficiency of visual selective attention: efficient visual search leads to inefficient distractor rejection. *Psychol. Sci.* 8 395–398 10.1111/j.1467-9280.1997.tb00432.x

[B35] Lavie N. Fox E. (2000). The role of perceptual load in negative priming. *J. Exp. Psychol. Hum. Percept. Perform.* 26 1038–1052 10.1037//0096-1523.26.3.1038 10884008

[B36] Lavie N. Hirst A. de Fockert J. W. Viding E. (2004). Load theory of selective attention and cognitive control. *J. Exp. Psychol. Gen.* 133 339–354 10.1037/0096-3445.133.3.339 15355143

[B37] Lavie N. Tsal Y. (1994). Perceptual load as a major determinant of the locus of selection in visual attention. *Percept. Psychophys.* 56 183–197 10.3758/BF03213897 7971119

[B38] Lewin G. R. Barde Y. A. (1996). Physiology of the neurotrophins. *Annu. Rev. Neurosci.* 19 289–317 10.1146/annurev.ne.19.030196.001445 8833445

[B39] Li T.-L. Gleeson M. (2004). The effect of single and repeated bouts of prolonged cycling and circadian variation on saliva flow rate, immunoglobulin A and alpha-amylase responses. *J. Sports Sci.* 22 1015–1024 10.1080/02640410410001716733 15801495

[B40] Lu B. Chow A. (1999). Neurotrophins and hippocampal synaptic transmission and plasticity. *J. Neurosci. Res.* 58 76–87 10.1002/(SICI)1097-4547(19991001)58:1<76::AID-JNR8>3.0.CO;2-0 10491573

[B41] McMorris T. Graydon J. (1997). The effect of exercise on cognitive performance in soccer-specific tests. *J. Sports Sci.* 15 459–468 10.1080/026404197367092 9386203

[B42] Moore R. D. Romine M. W. O’connor P. J. Tomporowski P. D. (2012). The influence of exercise-induced fatigue on cognitive function. *J. Sports Sci.* 30 841–850 10.1080/02640414.2012.675083 22494399

[B43] Neeper S. A. Gómez-Pinilla F. Choi J. Cotman C. (1995). Exercise and brain neurotrophins. *Nature* 373 109 10.1038/373109a0 7816089

[B44] North T. McCullagh P. Tran Z. V. (1990). Effect of exercise on depression. *Exerc. Sport Sci. Rev.* 18 379–414 10.1249/00003677-199001000-00016 2141567

[B45] Nybo L. Secher N. H. (2004). Cerebral perturbations provoked by prolonged exercise. *Prog. Neurobiol.* 72 223–261 10.1016/j.pneurobio.2004.03.005 15142684

[B46] Sato H. Takenaka I. Kawahara J. I. (2012). The effects of acute stress and perceptual load on distractor interference. *Q. J. Exp. Psychol.* 65 617–623 10.1080/17470218.2011.648944 22463388

[B47] Schachinger H. (2003). Cognitive and psychomotor function in hypoglycemia: response error patterns and retest reliability. *Pharmacol. Biochem. Behav.* 75 915–920 10.1016/S0091-3057(03)00167-9 12957236

[B48] Strachan M. W. Deary I. J. Ewing F. M. Ferguson S. S. Young M. J. Frier B. M. (2001). Acute hypoglycemia impairs the functioning of the central but not peripheral nervous system. *Physiol. Behav.* 72 83–92 10.1016/S0031-9384(00)00380-2 11239984

[B49] Swain R. Harris A. Wiener E. Dutka M. Morris H. Theien B. (2003). Prolonged exercise induces angiogenesis and increases cerebral blood volume in primary motor cortex of the rat. *Neuroscience* 117 1037–1046 10.1016/S0306-4522(02)00664-4 12654355

[B50] Sy J. L. Elliott J. C. Giesbrecht B. (2013). Post-perceptual processing during the attentional blink is modulated by inter-trial task expectancies. *Front. Hum. Neurosci.* 7:627 10.3389/fnhum.2013.00627 PMC3792353 24115924

[B51] Sy J. L. Guerin S. A. Stegman A. Giesbrecht B. (2014). Accurate expectancies diminish perceptual distraction during visual search. *Front. Hum. Neurosci.* 8:334 10.3389/fnhum.2014.00334 PMC4034704 24904374

[B52] Themanson J. R. Hillman C. H. (2006). Cardiorespiratory fitness and acute aerobic exercise effects on neuroelectric and behavioral measures of action monitoring. *Neuroscience* 141 757–767 10.1016/j.neuroscience.2006.04.004 16713115

[B53] Tomporowski P. D. Beasman K. Ganio M. S. Cureton K. (2007). Effects of dehydration and fluid ingestion on cognition. *Int. J. Sports Med.* 28 891–896 10.1055/s-2007-965004 17907076

[B54] van Praag H. Christie B. R. Sejnowski T. J. Gage F. H. (1999). Running enhances neurogenesis, learning, and long-term potentiation in mice. *Proc. Natl. Acad. Sci. U.S.A.* 96 13427–13431 10.1073/pnas.96.23.13427 10557337 PMC23964

